# 2-(4-Fluoro­phen­yl)-2*H*-chromen-4(3*H*)-one

**DOI:** 10.1107/S160053681105464X

**Published:** 2012-01-07

**Authors:** Michał Wera, Andriy G. Chalyi, Alexander D. Roshal, Jerzy Błażejowski

**Affiliations:** a Faculty of Chemistry, University of Gdańsk, J. Sobieskiego 18, 80-952 Gdańsk, Poland; b Institute of Chemistry, V.N. Karazin National University, Svobody 4, 61077 Kharkiv, Ukraine

## Abstract

In the crystal structure of the title compound, C_15_H_11_FO_2_, mol­ecules form inversion dimers through pairs of weak C—H⋯O hydrogen bonds. Dimers oriented in parallel, linked by C—H⋯π contacts, are arranged in columns along the *b* axis. The fluoro­phenyl ring and the benzene ring of the 2*H*-chromen-4(3*H*)-one unit are inclined to one another by 70.41 (16)°. They are respectively parallel in a given column or almost perpendicular [oriented at an angle of 87.8 (1)°] in neighbouring (inversely oriented) columns, forming a herringbone pattern.

## Related literature

For general background to flavanones, see: Grayer & Veitch (2006 ▶); Nijveldt *et al.* (2001 ▶). For related structures, see: Białońska *et al.* (2007*a* ▶,*b* ▶). For inter­molecular inter­actions, see: Novoa *et al.* (2006 ▶); Takahashi *et al.* (2001 ▶). For the synthesis, see: Aitmambetov & Kubzheterova (2002 ▶); Chen *et al.* (2011 ▶); Wera *et al.* (2010 ▶).
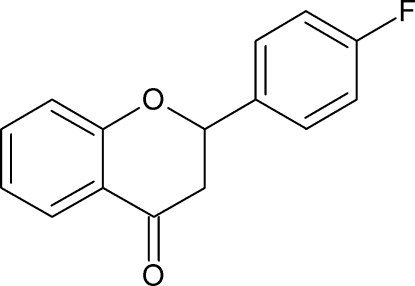


## Experimental

### 

#### Crystal data

C_15_H_11_FO_2_
*M* *_r_* = 242.24
Monoclinic, 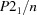

*a* = 11.7896 (13) Å
*b* = 5.2309 (8) Å
*c* = 19.740 (3) Å
β = 91.630 (11)°
*V* = 1216.9 (3) Å^3^
*Z* = 4
Mo *K*α radiation
μ = 0.10 mm^−1^
*T* = 295 K
0.6 × 0.05 × 0.05 mm

#### Data collection

Oxford Diffraction Gemini R Ultra Ruby CCD diffractometer
Absorption correction: multi-scan (*CrysAlis RED*; Oxford Diffraction, 2008 ▶) *T*_min_ = 0.919, *T*_max_ = 0.953
7630 measured reflections
2163 independent reflections
1080 reflections with *I* > 2σ(*I*)
*R*_int_ = 0.080

#### Refinement

*R*[*F* ^2^ > 2σ(*F* ^2^)] = 0.056
*wR*(*F* ^2^) = 0.160
*S* = 1.01
2163 reflections
164 parameters
H-atom parameters constrained
Δρ_max_ = 0.18 e Å^−3^
Δρ_min_ = −0.17 e Å^−3^

### 

Data collection: *CrysAlis CCD* (Oxford Diffraction, 2008 ▶); cell refinement: *CrysAlis RED* (Oxford Diffraction, 2008 ▶); data reduction: *CrysAlis RED*; program(s) used to solve structure: *SHELXS97* (Sheldrick, 2008 ▶); program(s) used to refine structure: *SHELXL97* (Sheldrick, 2008 ▶); molecular graphics: *ORTEP-3* (Farrugia, 1997 ▶); software used to prepare material for publication: *SHELXL97* and *PLATON* (Spek, 2009 ▶).

## Supplementary Material

Crystal structure: contains datablock(s) global, I. DOI: 10.1107/S160053681105464X/xu5408sup1.cif

Structure factors: contains datablock(s) I. DOI: 10.1107/S160053681105464X/xu5408Isup2.hkl

Supplementary material file. DOI: 10.1107/S160053681105464X/xu5408Isup3.cml

Additional supplementary materials:  crystallographic information; 3D view; checkCIF report

## Figures and Tables

**Table 1:** Hydrogen-bond geometry (Å, °) *Cg*1 is the centroid of the C5–C10 ring.

*D*—H⋯*A*	*D*—H	H⋯*A*	*D*⋯*A*	*D*—H⋯*A*
C2—H2⋯O11^i^	0.98	2.48	3.280 (4)	139
C3—H3*A*⋯*Cg*1^ii^	0.97	2.78	3.695 (3)	157

Symmetry codes: (i) 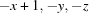; (ii) 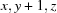.

**Fig. 1.**
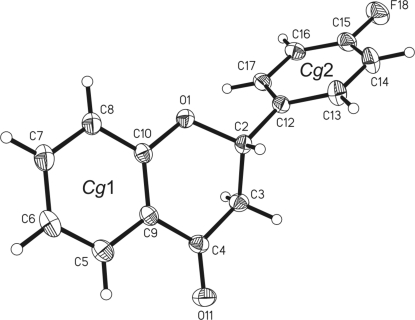
The molecular structure of the title compound showing the atom labeling scheme. Displacement ellipsoids are drawn at the 25% probability level, and H atoms are shown as small spheres of arbitrary radius.

**Fig. 2.**
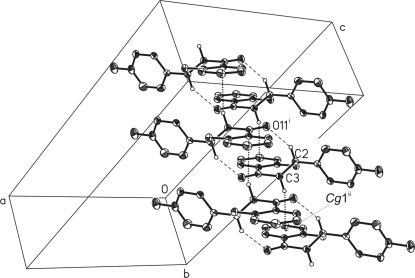
The arrangement of the molecules in the crystal structure. The C–H···O interactions are represented by dashed lines, the C–H···π contacts by dotted lines. H atoms not involved in interactions have been omitted. [Symmetry codes: (i) –x + 1, –y, –z; (ii) x, y + 1, z.]

**Fig. 3.**
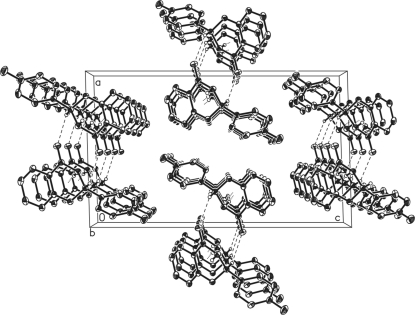
Columns in the crystal structure, viewed along the b axis. The C–H···O interactions are represented by dashed lines, the C–H···π contacts by dotted lines. H atoms not involved in interactions have been omitted.

## supplementary crystallographic information

### Comment

Flavanones [derivatives of 2-phenyl-2*H*-chromen-4(3*H*)-one] appear in numerous natural systems where they fulfil a beneficial role due to their antioxidant features (Nijveldt *et al.*, 2001; Grayer & Veitch, 2006). Here we present the structure of 2-(4-fluorophenyl)-2*H*-chromen-4(3*H*)-one, a flavanone that was obtained during the synthesis of a related flavonol (Wera *et al.*, 2010).

In the title compound (Fig. 1), the bond lengths and angles characterizing the geometry of the 2-phenyl-2*H*-chromen-4(3*H*)-one (flavanone) moiety are typical of this group of compounds (Białońska *et al.*, 2007*a*,*b*). With respective average deviations from planarity of 0.0103 (2)° and 0.0040 (2)°, the *Cg*1 and *Cg*2 benzene ring systems are oriented at a dihedral angle of 70.4 (1)° (in the case of 6-hydroxy-2-phenyl-2*H*-chromen-4(3*H*)-one and 2-(4-hydroxyphenyl)-2*H*-chromen-4(3*H*)-one this angle is equal to 74.4 (1)° (Białońska *et al.*, 2007*a*) and 74.8 (1)° (Białońska *et al.*, 2007*b*) respectively). The crystal structure data indicate that the 2*H*-chromen-4(3*H*)-one moiety is non-planar [average deviation from planarity: 0.1857 (2) mainly within the O1/C2–C4/C9/C10/O11 fragment, since the average deviation from planarity of the C5–C10 ring is 0.0103 (2)]. It is mainly the C2 atom that deviates from planarity, since the average deviation from planarity of the O1/C3–C10/C11 fragment is equal to 0.0368 (2).

In the crystal structure, the inversely oriented molecules form dimers through a pair of intermolecular C–H···O (Novoa *et al.*, 2006) interactions (Table 1, Fig. 2). Dimers oriented in parallel, linked by C–H···π (Takahashi *et al.*, 2001) contacts (Table 1, Fig. 2), are arranged in columns along the *b* axis (Fig. 3) that are dispersively stabilized in the crystal lattice. The adjacent *Cg*1 and *Cg*2 benzene rings are parallel in a given column or oriented at an angle of 87.8 (1)° in the two neighbouring, inversely oriented, columns, forming a herringbone pattern.

### Experimental

The title compound was synthesized following a procedure described elsewhere (Aitmambetov & Kubzheterova, 2002; Chen *et al.*, 2011). Briefly, 3-(4-fluorophenyl)-1-(2-hydroxyphenyl)prop-2-en-1-one was synthesized first by the condensation (with removal of H_2_O) of 1-(2-hydroxyphenyl)ethanone with 4-fluorobenzaldehyde in methanol/50% aqueous NaOH (1/1 *v*/*v*), then precipitated by neutralizing the reaction mixture with aqueous HCl, and finally separated by filtration (Wera *et al.*, 2010). The product thus obtained was subjected to cyclization in triethylamine/ethanol solution (by refluxing for 2–3 h). The reactant mixture was poured into cold HCl-acidified water which caused the precipitation of 2-(4-fluorophenyl)-2*H*-chromen-4(3*H*)-one. The filtered product was purified chromatographically (Silica Gel, chloroform/methanol, 20/1 *v*/*v*), and colorless crystals suitable for X-ray investigations were grown from absolute ethanol (m.p. = 351–353 K; lit. 352–353 K (Chen *et al.*, 2011)).

### Refinement

The H atoms of the C–H bonds were positioned geometrically, with C–H = 0.93 Å, 0.97Å and 0.98Å for the aromatic, methylene and methine H atoms respectively, and constrained to ride on their parent atoms with *U*_iso_(H) = *xU*_eq_(C), where *x* = 1.2 for aromatic H atoms and *x* = 1.5 for the methylene and methine H atoms.

### Crystal data

**Table:**

C_15_H_11_FO_2_	*F*(000) = 504
*M**_r_* = 242.24	*D*_x_ = 1.322 Mg m^−^^3^
Monoclinic, *P*2_1_/*n*	Mo *K*α radiation, λ = 0.71073 Å
Hall symbol: -P 2yn	Cell parameters from 2163 reflections
*a* = 11.7896 (13) Å	θ = 3.5–25.1°
*b* = 5.2309 (8) Å	µ = 0.10 mm^−^^1^
*c* = 19.740 (3) Å	*T* = 295 K
β = 91.630 (11)°	Needle, colorless
*V* = 1216.9 (3) Å^3^	0.6 × 0.05 × 0.05 mm
*Z* = 4	

### Data collection

**Table:**

Oxford Diffraction Gemini R Ultra Ruby CCD diffractometer	2163 independent reflections
Radiation source: Enhance (Mo) X-ray Source	1080 reflections with *I* > 2σ(*I*)
graphite	*R*_int_ = 0.080
Detector resolution: 10.4002 pixels mm^-1^	θ_max_ = 25.1°, θ_min_ = 3.5°
ω scans	*h* = −14→14
Absorption correction: multi-scan (*CrysAlis RED*; Oxford Diffraction, 2008)	*k* = −5→6
*T*_min_ = 0.919, *T*_max_ = 0.953	*l* = −18→23
7630 measured reflections	

### Refinement

**Table:**

Refinement on *F*^2^	Secondary atom site location: difference Fourier map
Least-squares matrix: full	Hydrogen site location: inferred from neighbouring sites
*R*[*F*^2^ > 2σ(*F*^2^)] = 0.056	H-atom parameters constrained
*wR*(*F*^2^) = 0.160	*w* = 1/[σ^2^(*F*_o_^2^) + (0.0555*P*)^2^] where *P* = (*F*_o_^2^ + 2*F*_c_^2^)/3
*S* = 1.01	(Δ/σ)_max_ < 0.001
2163 reflections	Δρ_max_ = 0.18 e Å^−^^3^
164 parameters	Δρ_min_ = −0.17 e Å^−^^3^
0 restraints	Extinction correction: *SHELXL97* (Sheldrick, 2008), Fc^*^=kFc[1+0.001xFc^2^λ^3^/sin(2θ)]^-1/4^
Primary atom site location: structure-invariant direct methods	Extinction coefficient: 0.021 (3)

### Special details

**Table:**

Geometry. All e.s.d.'s (except the e.s.d. in the dihedral angle between two l.s. planes) are estimated using the full covariance matrix. The cell e.s.d.'s are taken into account individually in the estimation of e.s.d.'s in distances, angles and torsion angles; correlations between e.s.d.'s in cell parameters are only used when they are defined by crystal symmetry. An approximate (isotropic) treatment of cell e.s.d.'s is used for estimating e.s.d.'s involving l.s. planes.
Refinement. Refinement of *F*^2^ against ALL reflections. The weighted *R*-factor *wR* and goodness of fit *S* are based on *F*^2^, conventional *R*-factors *R* are based on *F*, with *F* set to zero for negative *F*^2^. The threshold expression of *F*^2^ > σ(*F*^2^) is used only for calculating *R*-factors(gt) *etc*. and is not relevant to the choice of reflections for refinement. *R*-factors based on *F*^2^ are statistically about twice as large as those based on *F*, and *R*- factors based on ALL data will be even larger.

### Fractional atomic coordinates and isotropic or equivalent isotropic displacement parameters (Å^2^)

**Table:**

	*x*	*y*	*z*	*U*_iso_*/*U*_eq_	
O1	0.81543 (15)	0.0853 (4)	0.03640 (10)	0.0584 (6)	
C2	0.7340 (2)	0.1581 (6)	−0.01647 (15)	0.0559 (8)	
H2	0.7041	0.0020	−0.0379	0.067*	
C3	0.6364 (2)	0.3003 (6)	0.01423 (16)	0.0604 (9)	
H3A	0.6643	0.4583	0.0343	0.072*	
H3B	0.5811	0.3439	−0.0212	0.072*	
C4	0.5800 (3)	0.1446 (6)	0.06728 (16)	0.0580 (8)	
C5	0.6150 (3)	−0.1982 (6)	0.15344 (17)	0.0691 (10)	
H5	0.5387	−0.1907	0.1641	0.083*	
C6	0.6851 (4)	−0.3619 (7)	0.18806 (18)	0.0788 (11)	
H6	0.6563	−0.4688	0.2210	0.095*	
C7	0.7999 (3)	−0.3687 (7)	0.17398 (18)	0.0777 (11)	
H7	0.8482	−0.4776	0.1983	0.093*	
C8	0.8424 (3)	−0.2147 (6)	0.12410 (16)	0.0638 (9)	
H8	0.9194	−0.2177	0.1150	0.077*	
C9	0.6553 (3)	−0.0420 (5)	0.10249 (15)	0.0541 (8)	
C10	0.7694 (3)	−0.0553 (5)	0.08754 (15)	0.0533 (8)	
O11	0.48046 (18)	0.1698 (4)	0.08060 (13)	0.0784 (8)	
C12	0.7935 (2)	0.3105 (6)	−0.06892 (16)	0.0545 (8)	
C13	0.7726 (3)	0.2659 (7)	−0.13661 (19)	0.0813 (11)	
H13	0.7243	0.1333	−0.1498	0.098*	
C14	0.8221 (4)	0.4146 (8)	−0.1857 (2)	0.0939 (12)	
H14	0.8077	0.3834	−0.2315	0.113*	
C15	0.8924 (3)	0.6075 (8)	−0.1651 (2)	0.0766 (11)	
C16	0.9173 (3)	0.6583 (6)	−0.0993 (2)	0.0720 (10)	
H16	0.9666	0.7901	−0.0869	0.086*	
C17	0.8669 (2)	0.5075 (6)	−0.05082 (17)	0.0635 (9)	
H17	0.8826	0.5394	−0.0052	0.076*	
F18	0.94093 (19)	0.7554 (5)	−0.21355 (12)	0.1139 (9)	

### Atomic displacement parameters (Å^2^)

**Table:**

	*U*^11^	*U*^22^	*U*^33^	*U*^12^	*U*^13^	*U*^23^
O1	0.0545 (11)	0.0636 (13)	0.0569 (14)	0.0006 (10)	0.0003 (10)	0.0084 (11)
C2	0.0576 (18)	0.0598 (18)	0.050 (2)	0.0043 (15)	−0.0027 (15)	0.0036 (15)
C3	0.0594 (19)	0.0567 (18)	0.065 (2)	0.0072 (15)	0.0073 (16)	−0.0005 (16)
C4	0.0558 (19)	0.0593 (19)	0.059 (2)	−0.0006 (16)	0.0078 (16)	−0.0073 (16)
C5	0.079 (2)	0.069 (2)	0.060 (2)	0.0009 (19)	0.0113 (18)	−0.0054 (19)
C6	0.110 (3)	0.073 (2)	0.054 (2)	−0.004 (2)	0.016 (2)	0.0065 (18)
C7	0.101 (3)	0.072 (2)	0.060 (3)	0.011 (2)	−0.001 (2)	0.0089 (19)
C8	0.074 (2)	0.062 (2)	0.055 (2)	0.0056 (17)	−0.0002 (17)	0.0027 (17)
C9	0.065 (2)	0.0502 (17)	0.047 (2)	−0.0017 (15)	0.0066 (15)	−0.0043 (15)
C10	0.064 (2)	0.0491 (17)	0.0470 (19)	−0.0003 (15)	0.0034 (15)	−0.0043 (15)
O11	0.0585 (14)	0.0875 (17)	0.0899 (19)	0.0026 (12)	0.0133 (12)	0.0015 (14)
C12	0.0569 (18)	0.0570 (18)	0.050 (2)	0.0061 (15)	0.0045 (15)	−0.0055 (16)
C13	0.102 (3)	0.082 (3)	0.059 (3)	−0.010 (2)	−0.006 (2)	−0.001 (2)
C14	0.124 (3)	0.107 (3)	0.051 (3)	−0.010 (3)	0.001 (2)	0.011 (2)
C15	0.078 (2)	0.088 (3)	0.065 (3)	0.009 (2)	0.018 (2)	0.027 (2)
C16	0.068 (2)	0.073 (2)	0.077 (3)	−0.0060 (18)	0.018 (2)	0.005 (2)
C17	0.064 (2)	0.072 (2)	0.055 (2)	−0.0004 (18)	0.0090 (16)	−0.0048 (18)
F18	0.1241 (18)	0.1279 (19)	0.0916 (18)	0.0035 (15)	0.0346 (14)	0.0416 (15)

### Geometric parameters (Å, °)

**Table:**

O1—C10	1.373 (3)	C7—H7	0.9300
O1—C2	1.449 (3)	C8—C10	1.386 (4)
C2—C12	1.497 (4)	C8—H8	0.9300
C2—C3	1.512 (4)	C9—C10	1.388 (4)
C2—H2	0.9800	C12—C13	1.372 (4)
C3—C4	1.497 (4)	C12—C17	1.386 (4)
C3—H3A	0.9700	C13—C14	1.384 (5)
C3—H3B	0.9700	C13—H13	0.9300
C4—O11	1.217 (3)	C14—C15	1.361 (5)
C4—C9	1.479 (4)	C14—H14	0.9300
C5—C6	1.360 (5)	C15—C16	1.350 (5)
C5—C9	1.390 (4)	C15—F18	1.367 (4)
C5—H5	0.9300	C16—C17	1.387 (4)
C6—C7	1.389 (5)	C16—H16	0.9300
C6—H6	0.9300	C17—H17	0.9300
C7—C8	1.378 (4)		
			
C10—O1—C2	113.7 (2)	C7—C8—H8	120.3
O1—C2—C12	108.9 (2)	C10—C8—H8	120.3
O1—C2—C3	109.7 (2)	C10—C9—C5	118.5 (3)
C12—C2—C3	113.1 (2)	C10—C9—C4	120.4 (3)
O1—C2—H2	108.3	C5—C9—C4	121.0 (3)
C12—C2—H2	108.3	O1—C10—C8	116.9 (3)
C3—C2—H2	108.3	O1—C10—C9	122.4 (3)
C4—C3—C2	111.8 (2)	C8—C10—C9	120.7 (3)
C4—C3—H3A	109.3	C13—C12—C17	118.1 (3)
C2—C3—H3A	109.3	C13—C12—C2	120.6 (3)
C4—C3—H3B	109.3	C17—C12—C2	121.3 (3)
C2—C3—H3B	109.3	C12—C13—C14	121.2 (4)
H3A—C3—H3B	107.9	C12—C13—H13	119.4
O11—C4—C9	122.6 (3)	C14—C13—H13	119.4
O11—C4—C3	122.8 (3)	C15—C14—C13	118.2 (4)
C9—C4—C3	114.5 (3)	C15—C14—H14	120.9
C6—C5—C9	121.2 (3)	C13—C14—H14	120.9
C6—C5—H5	119.4	C16—C15—C14	123.2 (3)
C9—C5—H5	119.4	C16—C15—F18	118.5 (4)
C5—C6—C7	119.8 (3)	C14—C15—F18	118.3 (4)
C5—C6—H6	120.1	C15—C16—C17	117.8 (3)
C7—C6—H6	120.1	C15—C16—H16	121.1
C8—C7—C6	120.3 (3)	C17—C16—H16	121.1
C8—C7—H7	119.8	C12—C17—C16	121.4 (3)
C6—C7—H7	119.8	C12—C17—H17	119.3
C7—C8—C10	119.4 (3)	C16—C17—H17	119.3
			
C10—O1—C2—C12	179.8 (2)	C5—C9—C10—O1	177.6 (3)
C10—O1—C2—C3	55.5 (3)	C4—C9—C10—O1	−5.6 (4)
O1—C2—C3—C4	−57.2 (3)	C5—C9—C10—C8	−2.1 (4)
C12—C2—C3—C4	−179.0 (3)	C4—C9—C10—C8	174.7 (3)
C2—C3—C4—O11	−152.0 (3)	O1—C2—C12—C13	136.3 (3)
C2—C3—C4—C9	28.3 (4)	C3—C2—C12—C13	−101.4 (3)
C9—C5—C6—C7	2.0 (5)	O1—C2—C12—C17	−46.7 (3)
C5—C6—C7—C8	−1.5 (5)	C3—C2—C12—C17	75.5 (4)
C6—C7—C8—C10	−0.8 (5)	C17—C12—C13—C14	−0.7 (5)
C6—C5—C9—C10	−0.2 (5)	C2—C12—C13—C14	176.4 (3)
C6—C5—C9—C4	−177.0 (3)	C12—C13—C14—C15	−0.1 (6)
O11—C4—C9—C10	−177.1 (3)	C13—C14—C15—C16	0.9 (6)
C3—C4—C9—C10	2.6 (4)	C13—C14—C15—F18	−179.7 (3)
O11—C4—C9—C5	−0.3 (5)	C14—C15—C16—C17	−1.0 (5)
C3—C4—C9—C5	179.4 (3)	F18—C15—C16—C17	179.6 (3)
C2—O1—C10—C8	155.0 (3)	C13—C12—C17—C16	0.6 (5)
C2—O1—C10—C9	−24.7 (4)	C2—C12—C17—C16	−176.4 (3)
C7—C8—C10—O1	−177.1 (3)	C15—C16—C17—C12	0.2 (5)
C7—C8—C10—C9	2.6 (5)		

### Hydrogen-bond geometry (Å, °)

**Table:**

Cg1 is the centroid of the C5–C10 ring.

**Table:**

*D*—H···*A*	*D*—H	H···*A*	*D*···*A*	*D*—H···*A*
C2—H2···O11^i^	0.98	2.48	3.280 (4)	139
C3—H3A···Cg1^ii^	0.97	2.78	3.695 (3)	157

Symmetry codes: (i) −*x*+1, −*y*, −*z*; (ii) *x*, *y*+1, *z*.
